# The complete chloroplast genome sequence of *Lotus corniculatus* L.

**DOI:** 10.1080/23802359.2020.1860708

**Published:** 2021-01-17

**Authors:** Wenbo Jiang, Lin Ma, Dengxia Yi, Yongzhen Pang

**Affiliations:** Institute of Animal Science, Chinese Academy of Agricultural Sciences, Beijing, China

**Keywords:** Chloroplast genome, *Lotus corniculatus* L, Fabaceae

## Abstract

*Lotus corniculatus* L., a member of the Fabaceae family, is considered one of the most agriculturally important forage plants, owing to its anti-bloating properties; its ability to grow in low-fertility, acidic, and high-salinity soils; and high nutritional value. In this study, we obtained the complete chloroplast genome of *L. corniculatus* by Illumina sequencing and GetOrganelle assembly pipeline. The whole chloroplast genome of *L. corniculatus* is 150,700 bp in length, and has a typical circular structure with four parts: a large single-copy region (LSC 82,117 bp), a small single-copy region (SSC 18,275 bp), and a pair of inverted repeat regions (25,154 bp for both IRa and IRb). The overall GC content is 36.03%. The plastome has 109 unique genes, consisting of 78 protein-coding genes, 27 unique tRNA gene, and 4 unique rRNA genes. Based on the protein-coding gene sequences from 17 species, we reconstructed a maximum likelihood (ML) tree. The phylogenetic result shows that *L. corniculatus* has a closer relationship with *Lotus japonicas*.

*Lotus* species have a worldwide distribution and their circumscription is considered one of the most problematic issues within the *Loteae* taxonomy (Clifford and Grant 1993; Allan et al. 2003). Determining the phylogenetic relationship of *Lotus* species has been a challenge for taxonomic and evolutionary biologists because the phenotypes are quite similar in this genus. However, only one complete chloroplast genome from *Lotus* species has been reported (NC_002694) (Kato et al. 2000), and another complete chloroplast genome from *Lotus japonicus* ecotype B-129 was submitted to NCBI by early in the year of 2020 (AP022636). It is imperative to sequence more chloroplast genome of *Lotus* species. *Lotus corniculatus*, a member of genus *Lotus*, is considered one of the most agriculturally important forage crops, which is attributed by its anti-bloating properties; its ability to grow in low-fertility, acidic, and high-salinity soil; and high nutritional value (Sun et al. 2014). Here, we sequenced, assembled and annotated the complete chloroplast genome of *L. corniculatus*, which will provide useful perspectives to further clarify the evolutionary relationship in *Lotus* family.

Seeds of *L. corniculatus* were kept at the Forage Germplasm Bank at Institute of Animal Science of the Chinese Academy of Agricultural Sciences (Beijing, E116°29′, N40°03′). The voucher specimen (FR002) was deposited at the Herbarium of the department of Grassland, IAS-CAAS, Beijing, China. Total genomic DNA was extracted from the fresh leaves of *L. corniculatus* using DNAsecure Plant Kit (DP320-03, TIANGEN). The high-quality DNA was sheared to the fragments of 300 bp in length for the shotgun library construction. The sequencing was performed on the Illumina Novaseq PE150 platform (Illumina Inc, San Diego, CA), and 150 bp paired-end reads were generated. The filtered reads were assembled into the complete chloroplast genome using the program GetOrganelle v1.5 (Jin et al. 2020) with *L. japonicus* chloroplast genome (GenBank accession number: NC_002694) as a reference. The annotation of chloroplast genome was conducted through the online program CPGAVAS2 (Shi et al. 2019) and GeSeq (Tillich et al. 2017). The annotated genomic sequence has been registered into GenBank with the accession number (MT528596).

The complete chloroplast genome of *L. corniculatus* is 150,700 bp in length, which consists of a large single-copy region (LSC 82,117 bp), a small single-copy region (SSC 18,275 bp), and a pair of inverted repeat regions (25,154 bp for both IRa and IRb). The total GC content of *L. corniculatus* chloroplast genome is 36.03%, while the corresponding values of the LSC, SSC, IR regions are 33.69%, 29.64%, and 42.17%, respectively. A total of 128 genes, including 37 tRNA genes, 8 rRNA genes, and 83 protein-coding genes, are successfully annotated in the complete chloroplast genome sequence of *L. corniculatus*. The complete chloroplast genome contains 109 unique genes, including 78 protein-coding genes, 27 tRNA genes and four rRNA genes. Intron-exon structure analysis indicated that fifteen genes contain one intron (*atpF*, *ndhA*, *ndhB*, *rpoC*1, *petD*, *petB*, *rpl2*, *rps12*, *rp*s16, *trnI*, *trnV*, *trnT-CGU*, *trnA-UGC*, *trnK-UUU*, and *trnL-UAA*), and two genes (*ycf3* and *clpP*) have two introns. Five protein-coding genes (*ndhB*, *rps7*, *rpl2*, *rpl23* and *ycf2*), seven tRNA genes (*trnE-UUC*, *trnM-CAU*, *trnL-CAA*, *trnV-GAC*, *trnA-UGC*, *trnR-ACG* and *trnN-GUU*) and four rRNA genes (*rrn4.5*, *rrn5*, *rrn16*, and *rrn23*) are duplicated in the IR regions.

To investigate the phylogenetic relationship of *L. corniculatus* with other species in Fabaceae, we reconstructed a phylogenetic tree based on the protein-coding genes of 17 species. The sequences were aligned using MAFFT v7 (Katoh et al. 2017), and then the maximum likelihood tree (Figure 1) was constructed using RAxML-8.2.12 (Stamatakis 2014) with a bootstrap of 1000 repeats and PROTGAMMAILGX model, with *Vachellia seyal* (NC 036735) as outgroup. Phylogenetic analysis indicated that *L. corniculatus* was closely related to *L. japonicas*, which provides useful perspectives for further studying the evolutionary relationship in the *Lotus* family (Figure 1).

**Figure 1.**
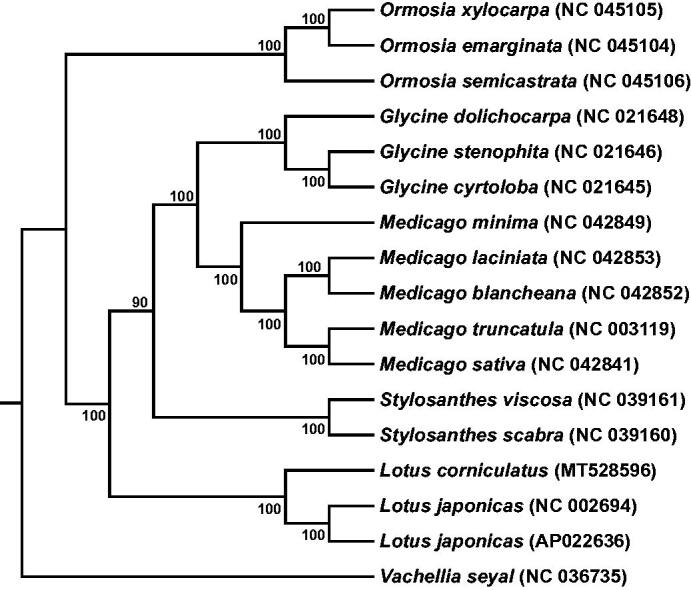
Phylogenetic tree reconstruction using maximum likelihood (ML) method based on a total of 17 complete chloroplast genome sequences of 17 species. Numbers above/below the branch lines represent ML bootstrap values.

## Data Availability

The data that support the findings of this study are openly available in NCBI at Genbank with accession number MT528596 (https://www.ncbi.nlm.nih.gov/nuccore/MT528596). Raw sequencing reads used in this study was deposited in the public repository SRA with accession number SRR12744821 (https://www.ncbi.nlm.nih.gov/sra/?term=SRR12744821).
